# The effect of interleukin-2 on canine peripheral nerve sheath tumours after marginal surgical excision: a double-blind randomized study

**DOI:** 10.1186/1746-6148-9-155

**Published:** 2013-08-08

**Authors:** Annika N Haagsman, Astrid C S Witkamp, Bart E Sjollema, Marja J L Kik, Jolle Kirpensteijn

**Affiliations:** 1 Department of Clinical Sciences of Companion Animals, Faculty of Veterinary Medicine, Utrecht University, Postbus 80154, 3508, TD Utrecht, The Netherlands; 2 Animal Medical Centre Amsterdam (AMCA), Isolatorweg 45, 1014AS Amsterdam, The Netherlands; 3 Department of Pathobiology, Pathology, Faculty of Veterinary Medicine, Utrecht University, Yalelaan1, 3584CL Utrecht, The Netherlands

**Keywords:** Sarcoma, Dog, rhIL2, PNST, Surgery, Intralesional, Injection

## Abstract

**Background:**

The objective of this study was to evaluate the effect on outcomes of intraoperative recombinant human interleukin-2 injection after surgical resection of peripheral nerve sheath tumours. In this double-blind trial, 40 patients due to undergo surgical excision (<5 mm margins) of presumed peripheral nerve sheath tumours were randomized to receive intraoperative injection of interleukin-2 or placebo into the wound bed.

**Results:**

There were no significant differences in any variable investigated or in median survival between the two groups. The median recurrence free interval was 874 days (range 48–2141 days), The recurrence-free interval and overall survival time were significantly longer in dogs that undergone the primary surgery by a specialist-certified surgeon compared to a referring veterinarian regardless of whether additional adjunct therapy was given.

**Conclusion:**

Overall, marginal excision of peripheral nerve sheath tumours in dogs resulted in a long survival time, but adjuvant treatment with recombinant human interleukin-2 (rhIL-2) did not provide a survival advantage.

## Background

Peripheral nerve sheath tumours (PNSTs) are spindle cell tumours that arise from the connective tissue components of the peripheral nerve and which can infiltrate the subcutis locally [1]. These tumours are thought to arise from perineural fibroblasts, which produce the non-myelinated connective tissues that surround the myelinated nerve fibre [2-6]. PNSTs appear to be pseudo-encapsulated, are locally invasive, and grow relatively slowly. Although PNST rarely metastasize, local recurrence is described commonly after resection [7-11].

Surgical removal of PNST with margins of minimally 2–3 cm margins in all dimensions is the therapy of choice. Marginal excision, which is defined as resection through the tumour pseudocapsule or surrounding reactive tissue, increases the likelihood of local recurrence and necessitates re-excision or postoperative radiation [12]. Since surgical removal alone does not guarantee complete eradication of the tumour, radiotherapy is advocated as adjuvant therapy, and especially when tumours are marginally or incompletely excised, to prolong survival and to decrease the incidence of recurrences. Radiotherapy can also be given preoperatively to decrease the size of the tumour [7,13]. Chemotherapy has also been used as adjuvant therapy for canine PNST with mixed results [7,13-15].

Immunotherapy has become popular in human medicine and decreases the rate of recurrence and metastases in a variety of tumours, including sarcomas [16-18]. Systemically administered cytokines, such as interleukin-2 (IL-2), activate the immune system to enhance immune-mediated responses against the tumour, thereby diminishing its metastatic potential [19]. IL-2 is a T-cell growth factor and induces clonal expansion of antigen-specific T-cells and activation of antigen-presenting cells (APC). It also increases the production of cytokines, such as interferon- gamma (IFN-γ), tumour necrosis factor alpha (TNF-α), and interleukin-6 (IL-6), and stimulates natural (NK) and lymphokine-activated killer cells (LAK) [11]. IL-2 modulates or promotes major histocompatibility complex (MHC) antigen expression, driving the tumour towards regression. In human cancers [20], recombinant human IL-2 (rhIL2) is commonly used as adjuvant to chemotherapy or radiotherapy [16,21], and its intratumoural administration has proven beneficial in melanoma and nasopharyngeal carcinoma, providing a complete response rate of 62.5% and a partial response rate of 21%; the rate of progressive disease was 16.5% [22].

Local rhIL2 therapy (intratumoural, peritumoural, or intravesical) is suggested to be more effective than systemic rhIL2 therapy and to have fewer side effects [17,20,23]. It has proven effective in the treatment of soft tissue sarcomas (STS) [22,23], malignant lymphoma [24], and gastrointestinal tumours [25] in humans, mice, and dogs. Local injection of rhIL2 leads to extravasation of erythrocytes at the injection site [26], causing stagnation of blood flow and leading to tumour cell death. At a later stage, leukocytes migrate to the dead tumour cells, forming a granuloma and causing angiogenesis [20]. Locally applied rhIL2 also decreases the size of distant metastases by an as yet unknown mechanism [24,26-28]. Local IL-2 therapy has been studied extensively in mice [29-32]. A study has reported its use in dogs with confirmed STSs, where treatment consisted of *Staphylococcus aureus* enterotoxin A and canine interleukin-2 (L_SEA/cIL-2) [33]. The treatment was well tolerated and had antitumour activity. Other tumours successfully treated with intratumoural rhIL2 therapy include canine transmissible venereal tumours [34], bovine ocular squamous cell carcinomas [35], equine sarcoid tumours [36], and canine mast cell tumours [37].

To the authors’ knowledge, the effect of adjuvant intralesional rhIL2 after surgical resection has not been studied in dogs. If rhIL2 has local or systemic effects, it should decrease the rates of recurrence and distant spread. The aim of this study was to evaluate the possible beneficial effect of local rhIL2 administration after marginal PNST removal in dogs.

## Methods

In the period between 2000 and 2003, 40 dogs (any breed, age, or sex) with a clinical diagnosis of PNST were referred to two referral practices in the Netherlands (Department of Clinical Sciences of Companion Animals, Faculty of Veterinary Medicine, Utrecht University [UU] and Animal Medical Centre Amsterdam [AMCA]). The inclusion criterion was presumed PNST that could not be removed with adequate 3-cm margins. This study included 21 dogs without previous surgery and 19 dogs with previous surgery. All the cases that were presented with previous surgery had macroscopic recurrences. Dogs with metastases or multiple tumours were excluded. The following data were collected: sex, age, weight, PNST localization (1. phalanges to carpus, 2 carpus to elbow, 3. elbow to shoulder, 4. cervical region, 5. phalanges to tarsus, 6. tarsus to stifle, 7. stifle to hip, 8. flank and 9. head), fine-needle biopsy and histology findings, location of metastasis and tumour grade. All dogs were screened for metastases, using right lateral, left lateral, dorsoventral or ventrodorsal radiographs of the lungs. A fine-needle aspiration biopsy (FNAB) was taken from all tumours. All tumours were removed with margins of 5 mm or less and submitted for histological examination by a certified pathologist (MK) to determine the type and grade of the tumour and the completeness of resection.

Recombinant human IL-2 (rhIL2; specific activity 18 × 10^6^ IU/mg; Proleukin® a gift from Chiron, Amsterdam, the Netherlands) was reconstituted to 1 mg/ml with distilled water. Polygeline was added to increase the stability of interleukin. Further dilutions were made with phosphate-buffered saline (PBS) supplemented with 0.1% bovine serum albumin, fraction V (BSA; Sigma Chemical Co., MO USA) [38]. The placebo contained distilled water with polygeline. Directly after surgical removal of the tumour, rhIL2 (1 ml containing 4.5 million IU) or placebo was injected evenly into the wound bed (0.01–0.05 ml per site) in an at random double-blinded fashion [20,34]. The wound bed was larger than the tumour by 5 mm in all dimensions and depended on the tension in that specific area. The exact wound size was not measured before or after injection. Surgery was performed by one of two surgeons (BS and JK). None of the patients received postoperative radiotherapy or chemotherapy. Blinding was broken after data had been accumulated and analysed.

Directly after surgery, the wounds were evaluated for signs of redness, haemorrhage, swelling, tenderness, and possible wound closure defects. All dogs were routinely screened for physical signs of regrowth and radiographic signs of metastasis at 1, 3, 6, and 12 months after surgery and every 12 months thereafter. If clinical signs developed before the screening time points, dogs were examined immediately. Recurrence was confirmed by evaluation of cytological or histological biopsy specimens. At the end point of the study, either the dog owner or the referring veterinarian was contacted by telephone for follow-up and administered a standard questionnaire including questions about breed, sex, age, disease-free interval, metastasis occurrence, tumour recurrence, survival time, and cause of death (if relevant). The investigators who administered the questionnaires were blinded to the treatment received.

Overall survival time (OS) was defined as the interval between the date of surgery and death due to the disease or the date on which the dog was last known to be alive. Metastasis free (MFI) and recurrence free (RFI) interval was defined as the interval between the date of the surgery and the date of either metastases or recurrence, or if there were no signs of recurrence or metastases, as the interval between surgery and the time on which the dog was last known to be alive. Dogs that had died from unrelated causes or which were still alive at the time of follow-up and without signs of either recurrence or metastases were considered censored. All dogs had a follow-up longer than 5 years. If a patient died without follow-up, the case was classified as lost to follow-up (LTF) and the last physical and diagnostic examination was used as end point. If a physical examination was performed but no diagnostic investigations, only RFI and OS were recorded; MFI was then coded as LTF. Owners were asked detailed questions about the surgical site, to determine whether RFI could be established; if there was any doubt, the date of the last physical examination was used in analyses.

### Statistical methods

A power analysis was performed in advance of the study, using survival data obtained from previous studies [8,11]. With an expected mean RFI difference of 20% per procedure and a variation coefficient of 15%, an α of 0.05, and a β of 0.15, a total sample size of 40 dogs was calculated.

Frequency distributions were calculated and categorical data were compared using Chi-square analysis. Fisher’s exact test was used when sample sizes were small, i.e. if more than 25% of the samples were smaller than 5. Normally distributed, continuous and interval categorical data were analysed using an analysis of variance (ANOVA). Logarithmic transformation was performed on variables that were not normally distributed. A hazard ratio (HR) of different variables on MFI, RFI, and OS was calculated using multivariate Cox proportional hazards analysis. P < 0.05 was considered significant. The Kaplan-Meier product limit method was used to estimate median RFI and OS. Group comparisons were made using the mantel-Cox log rank test (SPSS version 20.0). Statistical significance was defined as P < 0.05.

## Results

Marginal excision (<5 mm) was the only surgical option for all tumours. The dogs were of different breeds; 4 Beagles, 3 Boxers, 4 Bouviers des Flandres, 2 Bull Terriers, 8 cross breeds, 2 Fox terriers, 3 Bernese Mountain Dogs, 4 Labrador Retrievers, and one of each following breeds: English Bulldog, German shorthaired pointer, Leonberger, Husky, Belgian Shepherd Dog, German Shepherd Dog, White Shepherd Dog, Groenendaeler, Flatcoated Retriever, and Irish Wolfhound. Most were large-breed dogs; the median weight was 31 kg (range 12–51 kg). There were 23 male dogs and 17 female dogs. The median age of the dogs at referral was 9 years (range 3–14). Nineteen dogs had previously undergone tumour resection.

Four tumours were located on the forelimb in the area from the phalanges until the carpus, 15 from the carpus until the elbow, 7 from the elbow until the shoulder, 5 from the tarsus until the stifle, 4 from the stifle until the hip, 4 on the head, and 1 on the flank (Table 1). Most tumours were located at the antebrachium (38%) or the brachium (18%). Fine-needle aspiration biopsies (FNABs) were used to diagnose 13 mesenchymal proliferations and 15 mesenchymal tumours; the FNABs of 12 dogs were not diagnostic. There were 29 neurofibrosarcomas, 4 fibrosarcomas, 2 myxosarcomas, and 5 haemangiopericytomas. Tumour grade was determined to be low (grade 1) in 8 cases, medium (grade 2) in 20 cases, and high (grade 3) in 12 cases. The median tumour volume was 38.5 cm^3^ (range 1–2890 cm^3^). Six dogs developed metastatic disease in the lungs (5 dogs) and lymph nodes (1 dog). All tumours had dirty surgical margins (tumour reached or extended into the surgical margin).

**Table 1:** Statistical data of dogs with peripheral nerve sheath tumours receiving recombinant human interleukin-2 (rhIL2) or a placebo

	**rhIL2**	**Placebo**	**Significance**
Sex	12 M: 8F	10 M: 10 F	NS
Localisation	Front leg 10	Front leg 14	NS
	Rear leg 6	Rear leg 3	
	Axial 2	Axial 3	
Left vs right	L 9: R11	L14: R6	NS
Grade	Grade 1: 2	Grade 1: 6	NS
	Grade 2: 11	Grade 2: 9	
	Grade 3: 7	Grade 3: 5	
Previous sx	yes: no 10:10	yes: no 9:11	NS
Size*	45.6 ± 16.0	19.8 ± 4.0	NS
Age*	9.3 ± 0.55	8.9 ± 0.60	NS
Weight*	33.1 ± 1.7	29.2 ± 2.1	NS

*NS* No significant difference between the two groups, *vs* Versus, *sx* Surgery, *M* Male, *F* Female, *L* Left, *R* Right, *axial* Head or trunk; * described as mean ± standard error.

There was no significant difference between the control group and the IL-2 group in sex, age, weight, tumour localization, left versus the right side, radiographic appearance of the tumour, results of fine-needle biopsy, location of metastasis, and tumour grade (Table 1). There were also no between-group differences after exclusion of dogs with myxosarcomas and haemangiopericytomas, which are not classified as true PNSTs. The side effects of therapy were minimal and could not be distinguished from the normal side effects observed after skin surgery, such as minor redness, haemorrhage, tenderness of the wound, and wound swelling. No wound dehiscence was observed. There was no significant between-group differences in side effects (data not shown).

**Figure 1:**
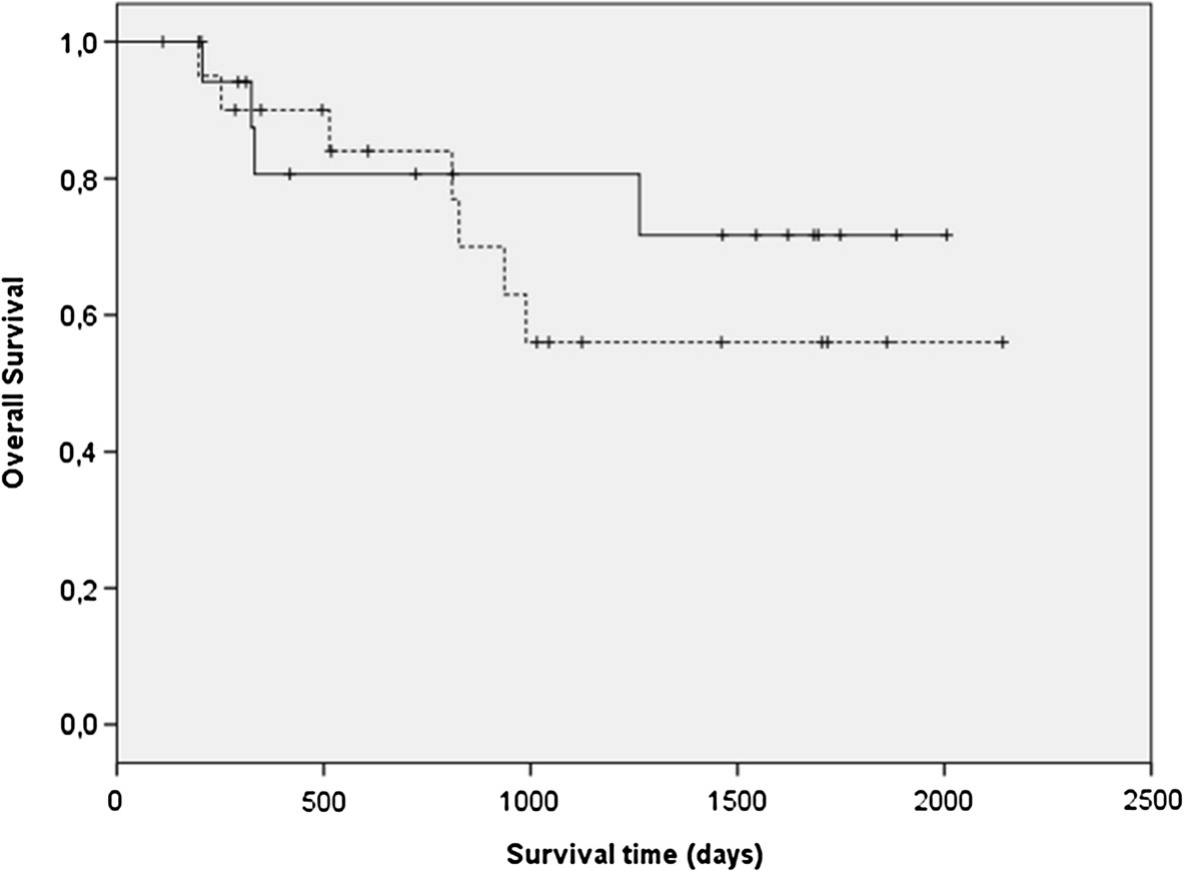
Kaplan-Meier survival curve of overall survival (OS) showing a non-significant difference between dogs with PNSTs treated with rhIL2 (dotted line) versus placebo (continuous line).

Overall, the median RFI was 874 days (range 48–2141 days), the median MFI was 1884 days (range 407–2141 days) and the median OS was not reached (range 197–2141 days). There was no significant difference in OS between the treatment groups (Figure 1) or by tumour group or grade. The rate of recurrence was 45% (9/20) in the rhIL2 group and 35% (7/20) in the placebo group. Six dogs developed metastatic disease, 5 in the lungs and 1 in the lymph nodes; all dogs had previously undergone surgery. Metastases developed in 3 dogs (15%) that had received placebo and in 3 dogs that had received rhIL2 and the incidence of metastases did not differ significantly between treatment groups. Of the 6 dogs with metastases, 2 were from grade 1 (n = 8), 3 from grade 2 (n = 20) and 1 from grade 3 tumors (n = 12). There was no significant difference in incidence of metastases between the three groups.

**Figure 2:**
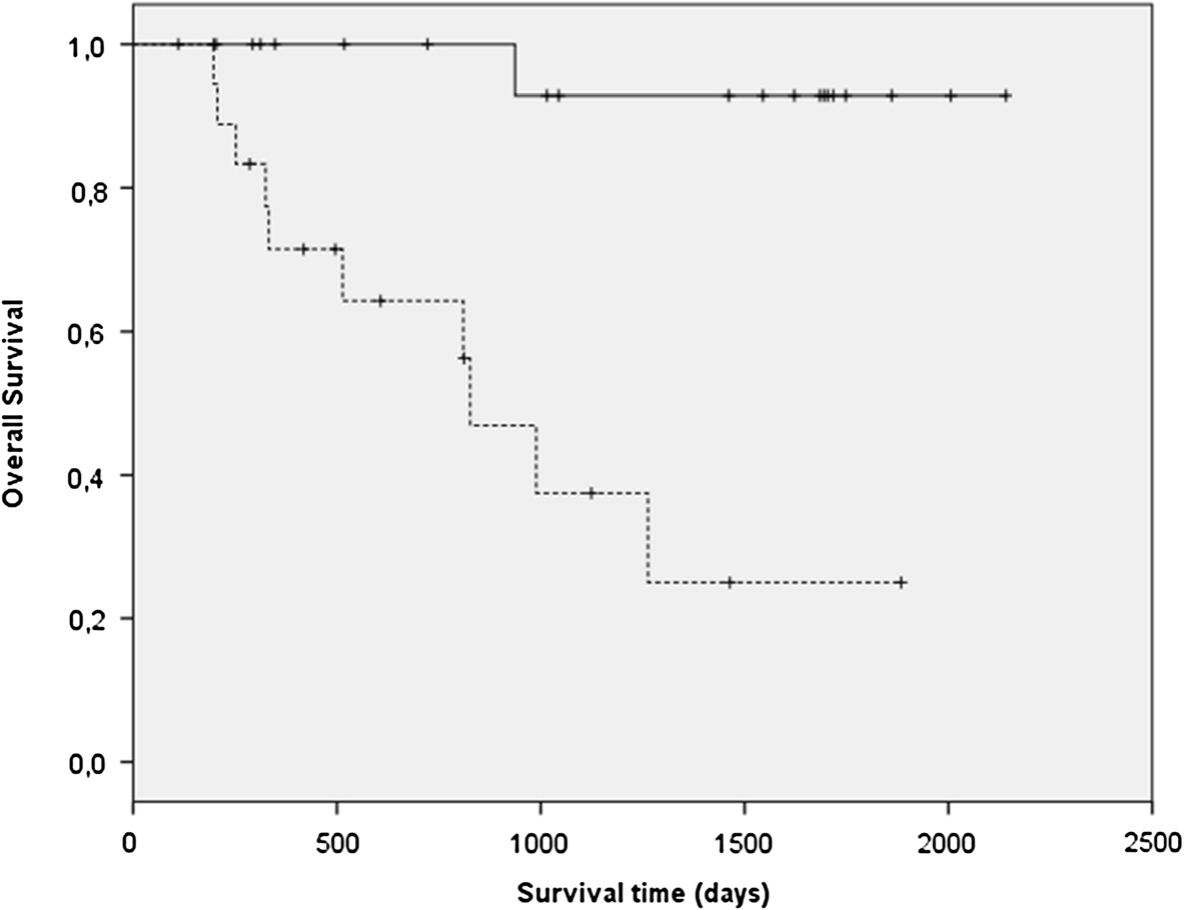
Kaplan-Meier survival curve of overall survival (OS) showing a significant difference (P < 0.001) between dogs with PNSTs that had undergone a previous surgery (dotted line) versus the ones that did not (continuous line).

**Figure 3:**
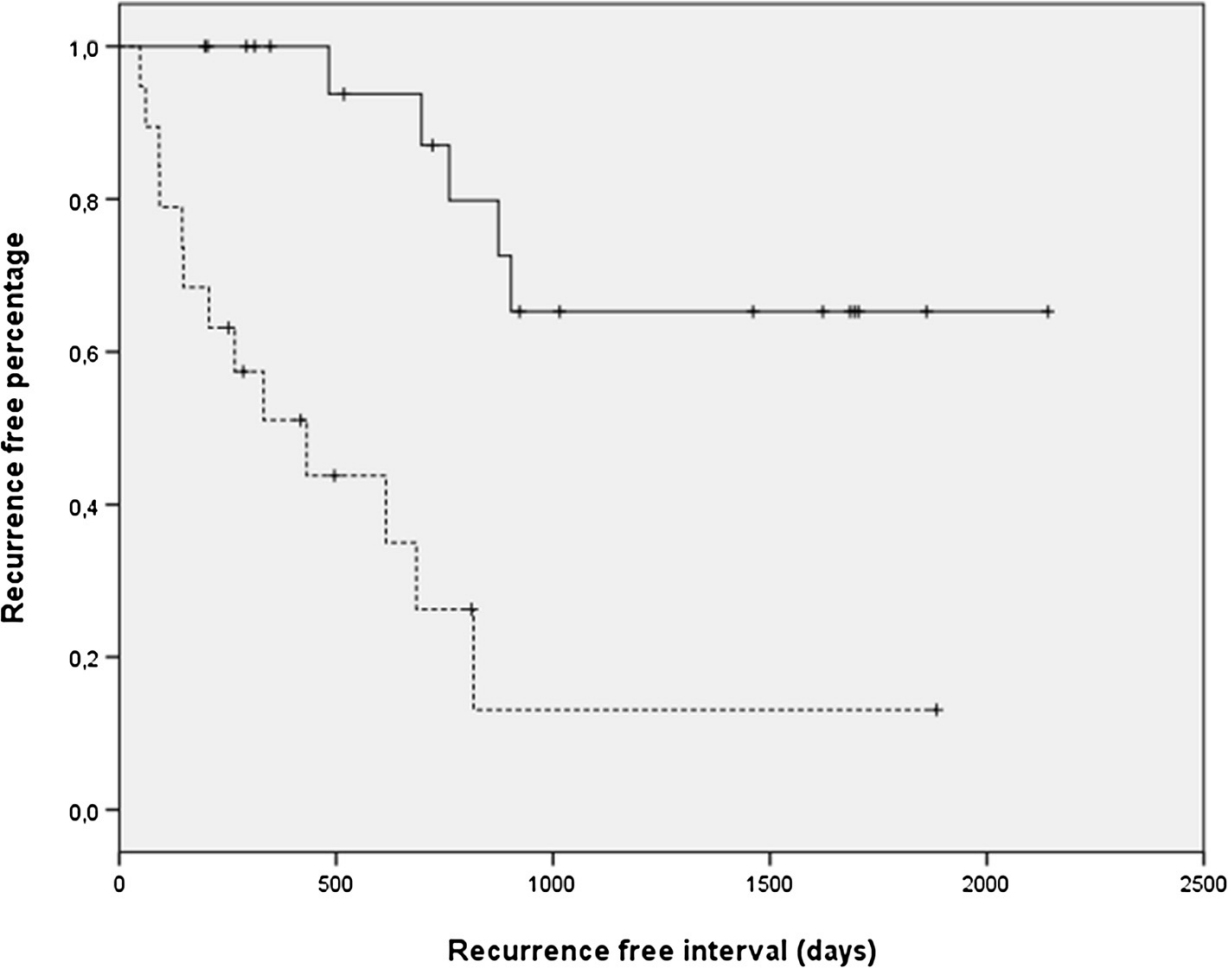
Kaplan-Meier survival curve of recurrence free interval (RFI) showing a significant difference (p < 0.001) between dogs with PNSTs that had undergone a previous surgery (dotted line) versus the ones that did not (continuous line).

The only significant difference in OS (P = 0.006) was found for dogs that had previously undergone surgery compared with those that had their first surgery at the referral clinics (UU or AMCA; Figure 2). Using multivariate analysis, the hazard ratio (HR) for recurrence was higher in dogs that had previously undergone surgery at the time of inclusion compared to dogs that had not had prior surgery (p < 0.001, HR 10.7, 95% CI 2.3-21.2; Figure 3). The only other factor that had a significant HR was the weight (P = 0.02, HR = 1.08, 95% CI 1.01-1.16). There was no significant difference in occurrence of metastases between dogs that had undergone previous surgery and the ones that had not, using multivariate analysis.

## Discussion

This study describes the effect on surgical outcomes of local injection of rhIL2 or placebo into the wound bed after surgical excision of PNST-like tumours. Treatment with rhIL2 did not significantly influence the MFI, RFI, or OS. In contrast, it has been reported to have a beneficial effect in various types of tumours in humans [16,17] and animals [25,29-31,35,36,39], providing a 6% complete response (CR) and 10% partial response (PR) in one study [16] or a 50% decrease in nasal angiosarcoma size in another [17]. In other studies, mice with inoculated lymphoma or mastocytoma treated intraperitoneally with IL-2 achieved a 70–90% CR [29-31], cattle with ocular squamous cell carcinoma treated with peritumoural IL-2 achieved a 69% CR [35], and horses with sarcoid tumours achieved a 14% PR and 43% CR with intratumoural IL-2 [36]. While systemic administration of IL-2 has also been used, locoregional administration strategies are more effective and successful [23,26]. Interestingly, local treatment is often effective systemically, eradicating metastases [32]. Systemic IL-2 therapy can lead to life-threatening toxicities and to hepatic dysfunction, which might result in hypotension and vascular fluid accumulation [23].

The use of IL-2 as sole adjuvant agent after sarcoma resection is not warranted at the moment. Additional therapies, such as chemotherapy or radiotherapy, may increase the efficacy of IL-2, but a large effect is unlikely, especially when a wide surgical excision is used, which is the preferred approach for PNSTs. In the above-mentioned study of horses with sarcoid tumours, local IL-2 treatment combined with chemotherapy (cisplatin) resulted in better outcomes than local IL-2 monotherapy (53% PR and 80% CR compared with 14% PR and 43% CR, respectively) [36].

Incomplete tumour resection increases patient morbidity, treatment costs, risk of further recurrence, and ultimately decreases survival time [40-43]. Therefore, the likelihood of recurrence is much lower if surgery is performed by a specialist-certified surgeon rather than a referring veterinarian [13]. We found a much lower recurrence rate in dogs that had not previously undergone surgery than in dogs that had previously undergone surgery. This difference can be explained in two ways: the veterinarians who removed the primary tumour might have used even smaller tumour margins than the specialist-certified surgeon or the previously removed tumour might have been more aggressive and more likely to recur. Of the 40 dogs, 8 had a grade 1 PNST, 20 a grade 2, and 12 a grade 3. There was no significant difference in grade of the removed PNST between the dogs with or without previous surgery. Nor did we find survival or recurrence rates to differ by tumour grade, but this might be a reflection of the relatively low number of cases.

The rate of recurrence was similar in the two treatment groups, 45% (9/20; rhIL2 group) versus 35% (7/20; placebo group). The overall recurrence rate (40%) was higher than the 15% reported in a comparable study of 41 dogs, in which all dogs that had surgery for recurrence had been referred after inadequate primary tumour resection [44]. This difference might be because we had a longer follow-up of minimally 5 years. Interestingly, tumour recurred at the site of excision many years after primary surgery, suggesting that dormant cells were present. This suggests that future studies should have longer follow-up times (>2 years) and that postoperative radiotherapy is warranted. Radiotherapy has been used in many studies after marginal resection of STS, with recurrence rates varying from 15% to 31%. Irradiated, incompletely resected STSs had a recurrence rate of 15% [42] and 17% [43]. However, because these studies did not investigate PNSTs and did not include rhIL2 therapy, we cannot compare results. Interestingly, a recent review of human cancer concluded that the response rate was higher when rhIL2 was used as adjuvant to radiotherapy [16], an approach that should be evaluated in the future. Alternative treatment options for incomplete tumour resection are re-excision of the wound bed with wider margins [40,42,44] and downstaging local disease with preoperative radiotherapy [40].

Most PNSTs in dogs have low metastatic rates. In this study, 15% of the PNSTs (6/40) had metastasized to lungs or lymph nodes, and rhIL-2 therapy did not influence the rate of metastasis. One study of IL-2 used in an adjuvant setting as local inhalation therapy for carcinoma reported that 2 of 7 treated dogs achieved full remission for more than a year (29%) [45]. Other canine STS studies have reported lower metastasis rates than the rate reported here. The rate of metastasis was 8% in a study in which radiotherapy was used as adjuvant therapy after incompletely resection [42]. The higher metastatic rate in our study might have been due to the longer follow-up, a more vigilant surveying system, or the presence of dormant cells in the tumour margins. The results of this study indicate that aggressive primary surgery is advisable for these tumours.

The higher incidence of metastasis may warrant the use of postoperative chemotherapy, a protocol not used in this study. However, the exact role of chemotherapy in preventing distant metastasis of STS is not known. Doxorubicin, ifosfamide, and mitoxantrone have been used in dogs and in humans. The overall response of canine sarcomas to doxorubicin was 23% in one study [46], but doxorubicin had no effect as adjuvant therapy in a recent study of high-grade STS [14]. Cisplatin applied in a biodegradable implant delivery system directly after marginal resection of STS in 19 dogs resulted improved survival in 9 dogs (47%) with a median follow-up of 874 days; 8 dogs died of tumour-unrelated causes, and 3 dogs had a recurrence (fatal in 1 dog) [47]. Recently, cyclophosphamide combined with piroxicam significantly prolonged the DFI of 30 dogs (median DFI >410 days) compared with that of 55 control dogs (median DFI 211 days) after marginal resection of STSs [43]. The median OS was not reached in our study, even though follow up times reached over 5 years. This compares favourable to other studies reported for surgery alone (1416 days) [41] or 2270 days after incomplete resection combined with adjuvant radiotherapy [42] and the 309 days reported in a study of intraoperative chemotherapy [47] or four-fraction palliative radiotherapy [48]. Chemotherapy is typically used in dogs with high-grade and incompletely resected tumours with a high metastatic rate, which might explain the high recurrence rate [13].

PNSTs on extremities often cannot be excised with wide margins and amputation of the limb is a suggested treatment alternative. However, marginal resection either as sole therapy or combined with radiotherapy may result in a similar long-term survival with less immediate morbidity. Marginal excision of low-grade (G_1_) STSs from the extremities of 35 dogs resulted in only 4 recurrences (11%) [49]. Similarly, a study showed a grade-dependent recurrence after marginal excision in 7% (3/41) of grade 1 (G_1_) tumours, 34% (14/41) of grade 2 (G_2_) tumours, and 75% (3/4) of grade 3 (G_3_) tumours [41]. Survival time was not influenced by grade in dogs treated with marginal excision [50]. Radical resection of STSs on extremities by limb amputation should therefore be considered as a last resort for recurrent and high-grade tumours, taking into account that the risk of metastases is higher in cases that had a recurrence [49,50]. A more aggressive approach, with preoperative incisional biopsies, seems warranted. Surgical margins can be smaller with lower grade tumours and still result in positive outcomes. The study reported here, had longer a follow up and a grade variation between groups that was identical but the comparison with historic data prevents any major conclusions when survival outcome is compared to the above-mentioned studies.

The study had several limitations. Although the entry criteria were strict and the double-blinded study had a well-executed follow-up regimen, a major limitation remains the compliance of owners. Unfortunately, owner compliance is never 100% and some owners lived too far away to bring their dogs to follow-up evaluations. In these cases, we consulted the referring veterinarian for follow-up information, but we have no way of knowing whether he/she had recently examined the patient. The variation in tumour size, location, and grade can also affect outcomes, especially in small case cohorts. In total, 4.5 million IU of rhIL2 was administered, but the area of distribution varied with the wound size, which limits extrapolation of the exact local dose of rhIL2. The size of the wound should have been measured prior to injection of rhIL2 or placebo to allow a better calculation of the exact dose per square cm^2^. Power analysis indicated that 40 dogs would be needed to detect a statistically significant difference between treatments. However, this is still a small number, especially when multiple observations and variables are used, such as the grade and histologic diagnosis. The use of two instead of one referral institute may have influenced the data although there were no significant differences between the two institutes for the variables examined (data not shown).

## Conclusion

The long-term prognosis of PNSTs was generally good, even after marginal resection. Wide resection of these tumours will most likely result in a better long-term RFI and OS for more malignant tumours, whereas marginal excision results in an increased recurrence rate and possible risk of metastases. This study showed that recurrences can occur years after the primary surgery, that recurrences are much more common after prior surgery, that metastases can occur in both lymph nodes and lungs, and that intralesional IL-2 does not confer a survival advantage in dogs with PNSTs. Future studies should use a stringent follow-up evaluation schedule as used in this study with minimally a 5-year follow-up to allow proper analysis of survival statistics. Although marginal excision of PNST (with or without adjuvant treatment with rhIL2) resulted in a long survival in a subset of dogs, presurgical incisional biopsy, wide excision, and/or adjuvant radiotherapy are advisable to prevent recurrence and possible metastases.

## Competing interests

The authors declare that they have no competing interests.

## Authors’ contributions

JK and BES were responsible for collecting patients and performed the surgeries. ANH and performed the analysis of the patients documents. MJLK performed the interpretation of the histology. ANH, JK and ACSW helped to draft the manuscript. All authors read and approved the final manuscript.
